# 2D-quantitative structure–activity relationships model using PLS method for anti-malarial activities of anti-haemozoin compounds

**DOI:** 10.1186/s12936-021-03775-2

**Published:** 2021-06-11

**Authors:** Phuong Thuy Viet Nguyen, Truong Van Dat, Shusaku Mizukami, Duy Le Hoang Nguyen, Farhana Mosaddeque, Son Ngoc Kim, Duy Hoang Bao Nguyen, Oanh Thi Đinh, Tu Linh Vo, Giang Le Tra Nguyen, Cuong Quoc Duong, Satoshi Mizuta, Dao Ngoc Hien Tam, M. Phuong Truong, Nguyen Tien Huy, Kenji Hirayama

**Affiliations:** 1 Faculty of Pharmacy, University of Medicine and Pharmacy At Ho Chi Minh City, Ho Chi Minh City, 700000 Viet Nam; 2 Department of Immunogenetics, Institute of Tropical Medicine (NEKKEN), Nagasaki University, 1-12-4 Sakamoto, Nagasaki, 852-8523 Japan; 3 Leading Programme, Graduate School of Biomedical Sciences, Nagasaki University, 1-12-4 Sakamoto, Nagasaki, 852-8523 Japan; 4 Center for Bioinformatics and Molecular Medicine, Graduate School of Biomedical Sciences, Nagasaki University, 1-14 Bunkyo, Nagasaki, Japan; 5 Asia Shine Trading & Service Co. Ltd., Ho Chi Minh City, 70000 Vietnam; 6 American University of the Carribean School of Medicine, 1 University Drive at Jordan Road, Cupecoy, Sint Maarten; 7 School of Tropical Medicine and Global Health, Nagasaki University, 1-12-4 Sakamoto, Nagasaki, 852-8523 Japan

**Keywords:** Antimalarial, Anti-haemozoin, In silico, Quantitative structure–activity relationship, QSAR

## Abstract

**Background:**

Emergence of cross-resistance to current anti-malarial drugs has led to an urgent need for identification of potential compounds with novel modes of action and anti-malarial activity against the resistant strains. One of the most promising therapeutic targets of anti-malarial agents related to food vacuole of malaria parasite is haemozoin, a product formed by the parasite through haemoglobin degradation.

**Methods:**

With this in mind, this study developed two-dimensional-quantitative structure–activity relationships (QSAR) models of a series of 21 haemozoin inhibitors to explore the useful physicochemical parameters of the active compounds for estimation of anti-malarial activities. The 2D-QSAR model with good statistical quality using partial least square method was generated after removing the outliers.

**Results:**

Five two-dimensional descriptors of the training set were selected: atom count (a_ICM); adjacency and distance matrix descriptor (GCUT_SLOGP_2: the third GCUT descriptor using atomic contribution to logP); average total charge sum (h_pavgQ) in pKa prediction (pH = 7); a very low negative partial charge, including aromatic carbons which have a heteroatom-substitution in “ortho” position (PEOE_VSA-0) and molecular descriptor (rsynth: estimating the synthesizability of molecules as the fraction of heavy atoms that can be traced back to starting material fragments resulting from retrosynthetic rules), respectively. The model suggests that the anti-malarial activity of haemozoin inhibitors increases with molecules that have higher average total charge sum in pKa prediction (pH = 7). QSAR model also highlights that the descriptor using atomic contribution to logP or the distance matrix descriptor (GCUT_SLOGP_2), and structural component of the molecules, including topological descriptors does make for better anti-malarial activity.

**Conclusions:**

The model is capable of predicting the anti-malarial activities of anti-haemozoin compounds. In addition, the selected molecular descriptors in this QSAR model are helpful in designing more efficient compounds against the P*. falciparum* 3D7A strain.

## Background

Malaria is a deadly infectious disease with about 228 million infected cases and 405,000 deaths worldwide, as recorded in 2018 [1]. The disease is caused by the bite of a mosquito having the *Plasmodium* parasite, which consists of five main species, *Plasmodium falciparum*, *Plasmodium vivax*, *Plasmodium ovale*, *Plasmodium knowlesi* and *Plasmodium malariae* [2]. Of these species, 90% of deaths (mostly in children) were related to *P. falciparum* [3]. Anti-malarial drugs, such as quinine, chloroquine, artemisinin, proguanil, pyrimethamine, mefloquine, and atovaquone, have been indicated as malaria treatment [4–6]. However, *Plasmodium* species developed resistance to most of these commonly used drugs. This resistance and the lack of a vaccine has become a major problem in malarial treatment in recent years [7]. Therefore, there is a pressing need to improve the efficiency by modifying existing compounds to face drug-resistance, as well as to discover novel anti-malarial compounds.

Due to funding investment constraints, in silico and collaborative approaches have become particularly attractive approaches for malaria drug discovery efforts. Some in silico techniques, namely molecular docking, pharmacophore models or quantitative structure–activity relationships (QSARs) significantly reduce the time and cost in the drug discovery process. Among the techniques, QSAR is considered a valuable tool that is applied extensively in rational drug design. The predictive QSAR model provides a mathematical correlation between the structural properties of the compounds and their anti-malarial activities using one-, two-, and three-dimensional descriptors of physicochemical properties, as well as structural characteristics relating to the activity. Once a reliable QSAR model has been developed, the biological activities of molecules can be predicted from the molecular descriptors by different methodologies, such as multiple linear regression (MLR), partial least squares (PLS), artificial neural networks (ANN) and heuristic method (HM). In recent years, QSAR models were applied to a variety of anti-malarial compounds to figure out physicochemical and structural characteristics that are essential for their activity [8–13]. Some QSAR models developed using sulfonamide and its derivatives, 5-(2-methylbenzimidazol-1-yl)-N-alklythiophene-2-carboxamid derivatives in order to select models that had the best predicting ability [6, 14]. Other studies used three-dimensional QSAR (3D-QSAR) combining with extra analysis gave striking structural characteristics that related to anti-malarial efficacy and the mechanism of action of anti-malarial compounds [15–17].

The anti-malarial activities of various groups of compounds, in particular quinine and its derivatives, had a satisfactory correlation with their anti-haemozoin activity [18]. Haemozoin is formed inside the food vacuoles of parasites to prevent lethal toxicity of haem, which is a product of the catabolism of haemoglobin. Thus, anti-haemozoin is an important therapeutic target in anti-malarial treatment. Recently, different approaches were highlighted. These approaches include the high-throughput screening (HTS) of anti-malarial drugs based on their physicochemical properties of haemozoin formation, or building computational models for in silico to screen novel anti-malarial drugs, or analog development from natural compounds or existing agents [19]. Of which, prediction models of correlation between anti-haemozoin and anti-malarial activities strongly assist in anti-malarial drug discovery, from modifying known compounds to identifying new chemical scaffolds for different targets of a large diverse database of compounds [18]. However, there is no QSAR model for anti-malarial activity of anti-haemozoin inhibitors. The aim of this study was to develop quantitative structure–activity relationship models to determine the influences of physiochemical structures of haemozoin inhibitors on anti-malarial activities.

## Methods

The best QSAR model will be chosen and could be applied for screening and designing better anti-haemozoin compounds for anti-malarial activities in next studies. QSAR modelling was conducted for anti-malarial activities of haemozoin inhibitors using the multiple linear regression (MLR) and partial least square (PLS) methods. Database of 21 compounds possessing both anti-malarial and anti-haemozoin activities were used for building QSAR models. The IC_50_ of these compounds varied from 0.06 – 10.5 µM (or pIC_50_ ranged between -1.02 to 1.22). The QSAR model was chosen based on the predicted fitness plots and statistical values of the models. Evaluation of QSAR models depended on three data sets, the training, validation and test sets. The results included the corresponding descriptors (coefficients) and correlation of the observed—predicted values of anti-malarial activities and the statistical parameters. The parameters, correlation coefficient or coefficient of determination (R^2^ or r-squared), cross-validated r^2^ (or Q^2^) and r^2^ for the external test set (R^2^_pred), and root mean square error (RMSE) as fitting criteria, were employed to evaluate the goodness of the models. The predictive model was tested based on different methods, such as internal for training set and external validation for test set, as well as Y-randomization method.

### Data set

To perform 2D-QSAR, a complete data set containing 21 anti-haemozoin compounds (Table 1) was taken from the experimental anti-malarial activities identifed in a previous work [20]. The half maximal inhibitory concentration (IC_50_) of the anti-haemozoin compounds was converted to logarithmic scale (pIC_50_) and used as the dependent variable. These compounds were randomly divided into two subsets, a training set (16 compounds) and a test set (6 compounds).

**Table 1:** Structures and their anti-malarial activities (IC_50_ values) of 21 anti-haemozoin compounds in building 2D-QSAR model

No	Compound	Structure	Anti-malarial activity (3D7A) IC_50_ (mM) ± SD	Anti-haemozoin activity IC_50_ (mM)
	C1		0.06	42.98
	C2		0.56 ± 0.27	18.3
	C3		1.01 ± 0.50	25.96
	C4		1.54 ± 0.07	53.44
	C5		3.06 ± 1.30	110
	C6		4.80 ± 1.70	198.1
	C7		6.80 ± 4.40	29.04
	C8		7.00 ± 1.40	43.98
	C9		8.00	4.58
	C10		8.00 ± 2.80	156
	C11		8.15 ± 2.60	34.67
	C12		8.95 ± 1.30	103.5
	C13		9.00 ± 1.40	14.01
	C14		9.00	36.16
	C15		9.00	160
	C16		9.26 ± 1.80	30.69
	C17		9.28 ± 2.40	28.5
	C18		9.50 ± 0.70	24.72
	C19		10.00 ± 1.40	41.18
	C20		10.00	38.54
	C21		10.50 ± 2.10	87.76

*IC*_*50*_ half maximal inhibitory concentration

### 2D-QSAR

**Fig. 1:**
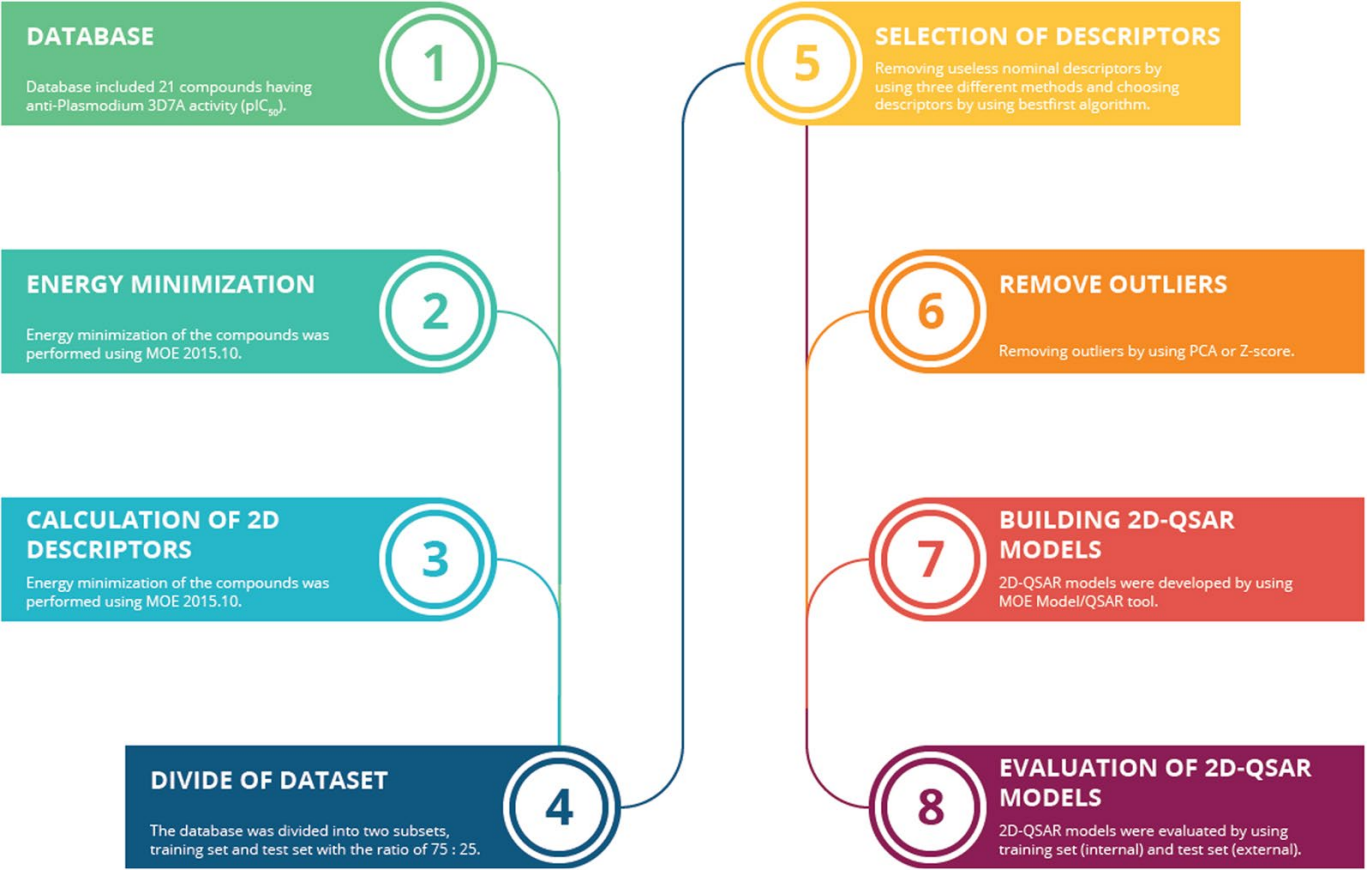
General steps of generating the QSAR model

A flowchart for developing 2D-QSAR was conducted following eight steps (Fig. 1). Initially, database included 21 compounds having anti-*Plasmodium* 3D7A activity. The IC_50_ values of these compounds were converted into logarithm scale logIC_50_ or pIC50 (pIC_50_ =− logIC_50_). The process of energy minimization of the compounds was performed using MOE 2015.10. A further step was the calculation of 2D descriptors. A total of 206 descriptors described molecular structures, including geometrical, physicochemical, sterical and lipophilic, which were calculated using Descriptors tool in MOE 2015.10. The database was subsequently divided into two subsets, a training set and a test set, with a 75:25 ratio. The database was divided randomly using RAND or *Diverse subset* using MOE. Selection of descriptors was carried out carefully. Some descriptors were removed based on three methods, firstly, if more than 15% compounds had descriptor values of 0 using Microsoft Excel. Secondly, using Rapidminer Studio 8.2.0 to take out of descriptors of the compounds which possess 50% similarity. Thirdly, remove randomly one of two descriptors having a cross correlation value of more than 70% using Rapidminer. These selected descriptors were also separated according the ratios of between 0 to 1 using Normalize in Rapidminer Studio based on the Eq. 1 below.

1 $${\text{X}}_{{\text{n}}} = \frac{{{\text{X}}_{0} - {\text{Min}}_{0} }}{{{\text{Min}}_{0} - {\text{Min}}_{0} }}$$

of which: ${\text{X}}_{{\text{n}}}$: Value; ${\text{X}}_{0}$: Initial value; ${\text{Min}}_{0}$,${\text{ Max}}_{0}$: Minimum, maximum of initial values.

Contigency tool in MOE and BestFirst—a searching method with assessment algorithm CfsSubsetEval in Weka 3.8.1 were used to find out the suitable descriptors.

Some outliers were removed by using PCA or Z-score, if the compounds had Z-score values of more than 2.0 before building 2D-QSAR. Using MOE with Model tool, 2D-QSAR models were developed using MLR. The best models were selected based on the highest values of the square of the coefficient of determination (R^2^) value, internally cross-validated R2 (Q^2^), and the external validated R^2^ (R^2^_pred). Of which, external validation used the test set while the training set was for model development. The internal validation parameters that were used, represented models’ goodness-of-fit and robustness. Finaly, evaluation of 2D-QSAR model on two datasets, training set and test set: Internal and external validations were conducted. The internal validation used the leave-one-out (LOO) cross-validation to internally validate the QSAR model. This is done by excluding the point(s) of training set data, then constructing the model based on the remaining data activities and finally, using this model to test the excluded data. This process was repeated until the training set activities were predicted. The coefficient of cross-validated R^2^ (or Q^2^) was calculated for the training set. The external validation was using the model for prediction of the biological activities of test set. The value of predicted correlation coefficient (R^2^_pred) value was calculated for the test set.

## Results and discussion

To conduct this study, database of 21 anti-haemozoin compounds was taken for building 2D-QSAR models (Table 1) to explore the structure–activity relationship of haemozoin inhibitors acting as anti-malarial agents. These compounds had in vitro anti-malarial activities against *P. falciparum* 3D7A and were used for QSAR modelling. The data set was randomly split into a training set (15 compounds) for model construction and test set (6 compounds), for validation of the model, respectively. The quality of a built QSAR model was demonstrated by the fitting and its predicting ability.

### Variable selection

Five two-dimensional descriptors of the training set were selected for QSAR modelling as they all had low inter-correlation (Table 2). They included atom count (a_ICM); + adjacency and distance matrix descriptor (GCUT_SLOGP_2: the third GCUT descriptor using atomic contribution to logP (using the Wildman and Crippen SlogP method) instead of partial charge); average total charge sum (h_pavgQ) in pKa prediction (pH = 7); a very low negative partial charge, including aromatic carbons which have a heteroatom-substitution in “ortho” position (PEOE_VSA-0) and molecular descriptor (rsynth: estimating the synthesizability of molecules as the fraction of heavy atoms that can be traced back to starting material fragments resulting from retrosynthetic rules). The study demonstrated that the average total charge sum (h_pavgQ) in pKa prediction (pH = 7) was the most important descriptor with the correlation coefficient values of about 0.41 (Table 2).

**Table 2:** Correlation matrix for inter-correlation of five selected descriptors and their correlation with the bioactivity (pIC_50_) against *P. falciparum* 3D7A with the Pearson’s correlation coefficient values

Descriptor type	Descriptor	a_ICM	GCUT_SLOGP_2	h_pavQ	PEOE_VSA-0	rsynth
Atom count	Atom information content (a_ICM)	1	0.0031	0.0385	0.0683	0.0647
Adjacency and distance Matrix	Using atomic contribution to logP (GCUT_SLOGP_2)	0.0031	1	0.0169	0.0001	0.0088
Average total charge sum	h_pavQ	0.0385	0.0169	1	0.0084	0.0447
Particle charge	PEOE_VSA-0	0.0683	0.0001	0.0084	1	0.0573
Molecular	Estimates the synthesizability of molecules (rsynth)	0.0647	0.0088	0.0447	0.0573	1
	pIC_50_	0.1327	0.1204	0.4059	0.0962	0.0733

IC_50_: half maximal inhibitory concentration

### QSAR model development

After selecting molecular descriptors, the linear QSAR models were built using the training set data. The outliers were checked and removed based on their values of PCA (principal component analysis), Z-score, and ZX-score of more than 2. There are four QSAR models that were developed based on the selection of different methods, namely PLS (Partial least squares) and PCR (Principal component regression), respectively with or without outliers (Table 3).

**Table 3:** Evaluation results of 2D-QSAR models generated by PLS (Partial least squares) and PCR (Principal component regression) methods

	Model 1 (PLS)					Model 2 (PCR)		
	With outliers		Without outliers			With outliers		Without outliers
Regression equation	pIC_50_ = 0.04406 – 1.17564 × a_ICM + 4.80603 × GCUT_SLOGP_2 + 0.46880 × h_pavgQ + 0.00334 * PEOE_VSA-0 + 0.29021 × rsynth		pIC_50_ = − 4.90988 + 1.98542 × a_ICM + 0.74756 × GCUT_SLOGP_2 + 0.59815 × h_pavgQ + 0.00837 × PEOE_VSA-0 – 0.12277 × rsynth			pIC_50_ = 0.04406 – 1.17564 × a_ICM + 4.80603 × GCUT_SLOGP_2 + 0.46880 × h_pavgQ + 0.00334 × PEOE_VSA-0 + 0.29021 × rsynth		pIC_50_ = −4 .95576 + 2.02008 × a_ICM + 0.19451 × GCUT_SLOGP_2 + 0.56938 × h_pavgQ + 0.00850 × PEOE_VSA-0 – 0.01478 × rsynth
R^2^	0.587642		0.745031			0.587642		0.738223
RMSE	0.392729		0.166261			0.392729		0.168465
Q^2^	0.025752		0.316410			0.025752		0.317759
R^2^_pred	0.773600		0.955400			0.773600		0.954200

### Validation of QSAR models

The evaluation of the QSAR models included the internal and external validations. The parameters for internal validation were R^2^ (a correlation coefficient), Q^2^ (predictive ability of the built QSAR models in the training set data employing leave-one-out (LOO) cross-validation method), and R^2^_pred (predictive ability for the test set). QSAR model is selected if it complies with the three criteria: the values of the high correlation coefficient (R^2^) between the experimental and the predicted values, the predictive ability of the model for the training set Q^2^ > 0.5, and the low standard deviation (RMSE). The comparison of four generated 2D-QSAR models were evaluated and compared in Table 3. The results showed that the QSAR models gave similar evaluation results by using PLS or PCR methods with outliers. This means that using different methods for the whole training dataset did not affect the development of the QSAR models. However, after removing outliers, the PLS model gave the better results, and the PCR model without outliers was worst than the others (Table 3). Therefore, the best QSAR model was the PLS model without outliers. The regression equation is represented as following:

$${\rm{pI}}{{\rm{C}}_{50}} = {\rm{ }} - 4.90988{\rm{ }} + {\rm{ }}1.98542{\rm{ }} \times {\rm{ a}}\_{\rm{ICM }} + {\rm{ }}0.74756{\rm{ }} \times {\rm{ GCUT}}\_{\rm{SLOGP}}\_2{\rm{ }} + {\rm{ }}0.59815{\rm{ }} \times {\rm{ h}}\_{\rm{pavgQ}}~ + {\rm{ }}0.00837{\rm{ }} \times {\rm{ PEOE}}\_{\rm{VSA}} - 0{\rm{ }}{-}{\rm{ }}0.12277{\rm{ }} \times {\rm{ rsynth}}.$$

where: R^2^ = 0.745031, RMSE = 0.166261, Q^2^ = 0.316410, and R^2^_pred = 0.9554.

The high values of R^2^ = 0.745; low standard error (RMSE = 0.166) and the good predictive ability: R^2^__Pred_ = 0.9554 (for the test set) indicated suitability of the model for predicting the anti-malarial activities of other haemozoin inhibitors from the existing anti-haemozoin compounds (Table 3). The experimental or observed versus predicted amounts of pIC_50_ of haemozoin inhibitors as anti-malarial structures against 3D7A strain were presented in Table 4 and Fig. 1. As can be seen in the Table 4, the predicted values of pIC_50_ values were in good agreement with the values of experimental pIC_50_.

**Table 4:** The values of selected descriptors and observed/predicted activities (pIC_50_)

No	Compound identification	Structure	Antimala IC_50_ (mM) ± SD	Anti-hemIC_50_ (mM)	Experimental pIC_50_	Predicted pIC_50_	a_ICM	GCUT_SLOGP_2	h_pavgQ	PEOE_VSA-0	Rsynth
1.	C1**		0.06	42.98	1.2185	− 0.006	1.53764	0.22929	1.98287	70.91325	0.81250
2.	C2**		0.56 ± 0.27	18.3	0.25181	− 1.573	1.48663	0.17043	0.03097	36.76471	0.55555
3.	C3		1.01 ± 0.50	25.96	− 0.00432	− 0.256	1.71097	0.13338	0.98667	77.47996	0.66666
4.	C4		1.54 ± 0.07	53.44	− 0.18752	− 0.303	1.50003	0.14600	0.98813	123.27970	0.84000
5.	C5		3.06 ± 1.30	110	− 0.48572	− 0.382	1.70074	0.16534	-0.00275	129.77250	0.46154
6.	C6		4.80 ± 1.70	198.1	− 0.68124	− 0.804	1.64727	0.05872	0.79449	43.25748	0.37037
7.	C7		6.80 ± 4.40	29.04	− 0.83251	− 0.767	1.68854	0.18846	0.78682	33.21112	0.80769
8.	C8		7.00 ± 1.40	43.98	− 0.84510	− 0.840	1.62957	0.13516	0.00209	98.03923	0.71875
9.	C9		8.00	4.58	− 0.90309	− 1.007	1.88074	0.19433	− 0.55864	49.01962	0.42857
10.	C10*		8.00 ± 2.80	156	− 0.90309	− 0.994	1.71613	0.13718	0.00000	51.22816	0.18518
11.	C11*		8.15 ± 2.60	34.67	− 0.91116	− 1.509	1.61260	0.13754	0.00052	24.50981	0.88889
12.	C12**		8.95 ± 1.30	103.5	− 0.95182	− 1.481	1.38153	0.15146	0.25559	58.58442	0.57143
13.	C13		9.00 ± 1.40	14.01	− 0.95424	− 0.760	1.79430	0.13340	0.18358	57.32159	0.83333
14.	C14*		9.00	36.16	− 0.95424	− 1.180	1.58409	0.15569	0.01251	65.69160	0.72727
15.	C15		9.00	160	− 0.95424	− 0.608	1.66637	0.08536	0.93749	51.42806	0.50000
16.	C16**		9.26 ± 1.80	30.69	− 0.96661	− 0.415	1.85538	0.21564	0.96585	12.25490	0.25000
17.	C17*		9.28 ± 2.40	28.5	− 0.96755	− 0.968	1.66447	0.16562	0.17235	61.27452	0.83333
18.	C18*		9.50 ± 0.70	24.72	− 0.97772	− 1.291	1.59500	0.17886	0.07861	37.01379	0.31250
19.	C19		10.00 ± 1.40	41.18	− 1.00000	− 1.164	1.76194	0.13516	0.05321	24.50981	0.73913
20.	C20*		10.00	38.54	− 1.00000	− 1.298	1.66327	0.09772	0.08231	27.65577	0.36364
21.	C21		10.50 ± 2.10	87.76	− 1.02119	− 0.979	1.64467	0.16386	0.00702	70.19921	0.40000

^*^Compounds belong to test set

**Outlier compounds

**Fig. 2:**
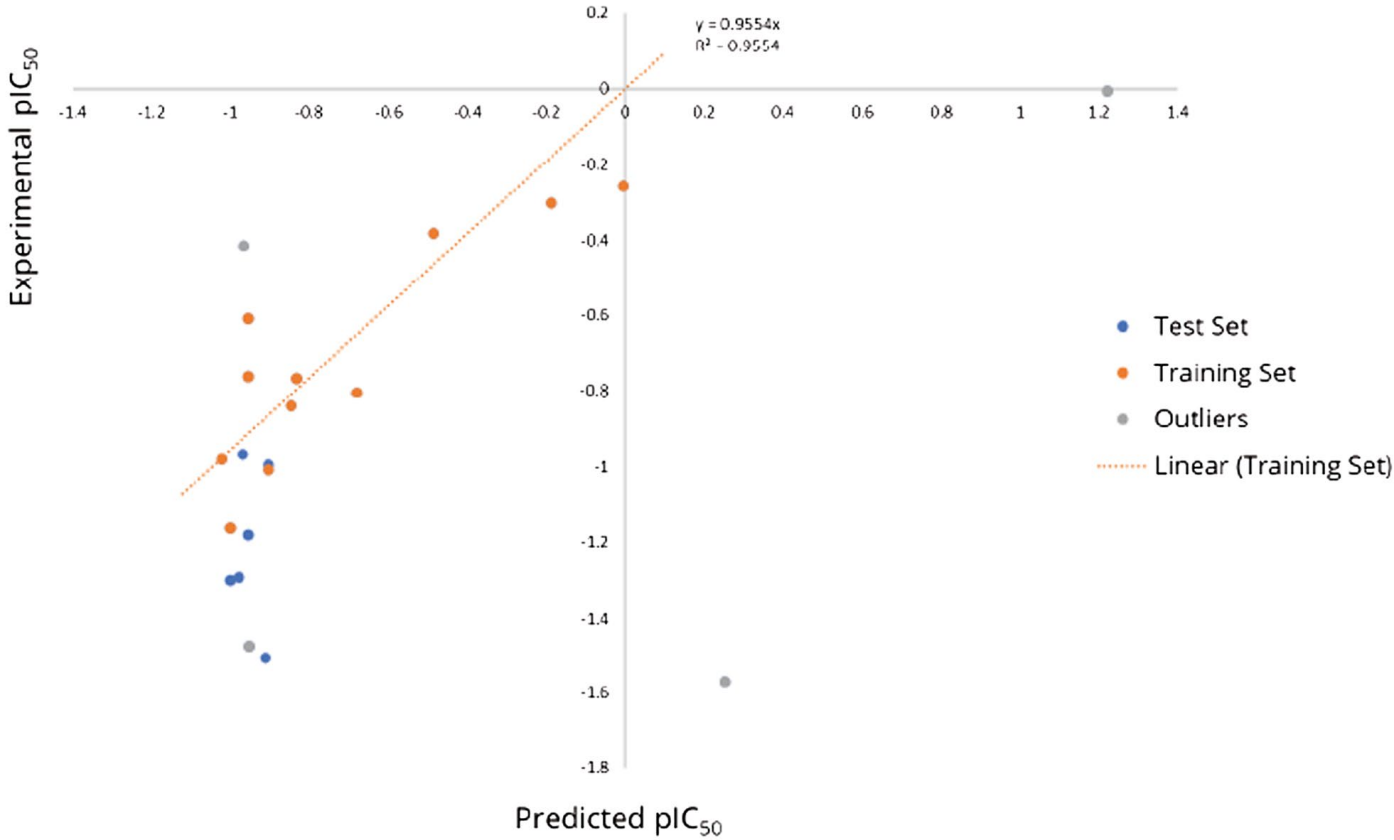
Plot of the correlation between the experimental pIC_50_ and the pIC_50_ predicted anti-malarial activities using partial least squares model

The linear graphical plot was depicted in Fig. 2. The graph illustrated the good overlap of the observed and predicted activities of the data set with the high of correlation coefficient of R^2^ = 0.9554 (Fig. 2). The predicted values of pIC_50_ varied between − 1.021 to 1.222 with the value ranges of the selected descriptors presented in Table 5. The decrease of these descriptors led a decrease of pIC_50_ values meaning the increase of IC_50_ which is a decrease of anti-malarial activities.

**Table 5:** Values ranges of selected descriptors in 2D-QSAR model

	a_ICM	GCUT_SLOGP_2	h_pavQ	PEOE_VSA-0	rsynth
Min	1.500031	0.058723	− 0.558641	24.509808	0.370370
Max	1.880740	0.194329	0.988134	129.772461	0.839999

### Interpretation of descriptors

It was clearly inferred that the average total charge sum (h_pavgQ) in pKa prediction (pH = 7) contributed the most to the values of pIC_50_, which could be used as one indicator for predicting anti-malarial activities of other anti-haemozoin agents. The higher average total charge sum (h_pavgQ) in pKa prediction (pH = 7) resulted in increasing values of pIC_50_, or decreasing of IC_50_, indicating better anti-malarial activities (Table 4). The positive sign of these descriptors indicated that the larger the value of pIC_50_, the lower IC_50_ of the compound. In addition, this feature was also taken for evaluation and prediction of anti-malarial activities for some anti-malarial drugs, such as quinine, pyrimethamine, halofantrine and mefloquine. It was found that the higher their calculated h_pavgQ values, the better anti-malarial activities.

Furthermore, the decrease of distance matrix descriptor (GCUT_SLOGP_2) or the third GCUT descriptor using atomic contribution to logP could lead better anti-malarial activity. The result was compatible with the previous study as this descriptor represents for lipophilicity and low lipophilicity, especially at pH 3, 4, and 5 were significantly related to better anti-malarial activity of anti-haemozoin molecules.

In addition, the positive sign of the PEOE_VSA-0 descriptor, a very low negative partial charge, including aromatic carbons which have a heteroatom-substitution in “ortho” position suggests that increasing in the PEOE_VSA-0 will decrease the inhibitory potency of anti-haemozoin compounds. The increase of atom count (a_ICM), topological descriptors or structural components of the molecules have an effect on the variation of anti-malarial inhibitory activity of the anti-haemozoin compounds. Moreover, molecular descriptor (rsynth: estimating the synthesizability of molecules as the fraction of heavy atoms that can be traced back to starting material fragments resulting from retrosynthetic rules) was the least contributive. In addition, the predicted pIC_50_ in Table 4 were much different with the experimental pIC_50_ values for the outliers, especially C1, C2, C12, C16. As a result, removing these outlier compounds from the training set for building QSAR model was essential.

The limitation of this study is the toxicity evaluation. In fact, there is no model predicting both the structure–activity and the structure–toxicity relationships, but they are separate models either predicting the structure–activity or the structure toxicity. Therefore, this QSAR model is not suitable for predicting the toxicity of the compounds. Another QSAR model for toxicity is required.

## Conclusion

With the 15 anti-haemozoin compounds, the satistically satisfactory 2D-QSAR model using PLS method was generated after removing the outliers. Five two-dimensional descriptors of the training set were selected: atom count (a_ICM); adjacentcy and distance matrix descriptor (GCUT_SLOGP_2: the third GCUT descriptor using atomic contribution to logP; average total charge sum (h_pavgQ) in pKa prediction (pH = 7); a very low negative partial charge, including aromatic carbons which have a heteroatom-substitution in “ortho” position (PEOE_VSA-0) and molecular descriptor (rsynth: estimating the synthesizability of molecules as the fraction of heavy atoms that can be traced back to starting material fragments resulting from retrosynthetic rules), respectively. The interpretation of the developed model suggests that the anti-malarial activity of haemozoin inhibitors increases with molecules having higher average total charge sum (h_pavgQ) in pKa prediction (pH = 7). The QSAR model also highlights that the descriptor using atomic contribution to logP or the distance matrix descriptor (GCUT_SLOGP_2), and structural component of the molecules, including topological descriptors does make for better anti-malarial activity.

## Data Availability

The datasets used and/or analysed during the current study are available from the corresponding author on reasonable request.

## Abbreviations

ANN: Artificial neural networks
HM: Heuristic method
HTS: High-throughput screening
LOO: Leave-one-out
MLR: Multiple linear regression
PLS: Partial least squares
QSAR: Quantitative structure–activity relationship
